# The influence of research self-efficacy and learning engagement on Ed.D students’ academic achievement

**DOI:** 10.3389/fpsyg.2025.1562354

**Published:** 2025-06-02

**Authors:** Haifei Miao, Rong Guo, Ming Li

**Affiliations:** School of Education, Shaanxi Normal University, Xi’an, China

**Keywords:** research self-efficacy, learning engagement, academic achievement, doctor of education, doctoral education

## Abstract

**Introduction:**

The doctor of education (Ed.D) degree plays a crucial role in developing highly specialized professionals in the field of education. Previous researches have shown that research self-efficacy is a positive psychological factor that enhances students’ academic performance. However, there is limited research specifically addressing how to improve the academic achievement of Ed.D students in higher education.

**Methods:**

Grounded in social cognitive theory, this study aims to examine the predictive power of research self-efficacy and the mediating role of student engagement in the academic achievement of Ed.D students. A total of 310 participants were included in this study. Pearson correlation coefficients were used to explore the relationships between research self-efficacy, learning engagement, and academic achievement. Mediation analysis was conducted to assess the indirect effect of student engagement on the relationship between research self-efficacy and academic achievement.

**Results:**

The findings revealed significant and positive correlations among research self-efficacy, learning engagement, and academic achievement. Research self-efficacy was found to directly predict academic achievement and also to indirectly influence it through the mediating effect of student engagement. Learning engagement refers to the degree of effort, persistence, concentration, problem-solving strategies, and affective connection that a student demonstrates during learning activities, encompassing cognitive, emotional, and behavioral dimensions. That is, the higher the Ed.D students’ research self-efficacy, the more they invest in cognitive, emotional and behavioral engagement, actively participating in learning and research activities, which in turn enhances their academic achievement.

**Discussion:**

These results provide valuable insights into the mechanisms by which research self-efficacy influences academic achievement in Ed.D students, highlighting the importance of learning engagement. The study’s findings have implications for designing interventions aimed at enhancing Ed.D students’ academic success.

## 1 Introduction

The doctor of education (Ed.D) is a professional degree designed to cultivate highly specialized professionals in education, teaching, and educational leadership. As a professional doctorate, the Ed.D prepares educators to apply specific practices, generate new knowledge, and contribute to the stewardship of the education profession (The Carnegie Project on the Education Doctorate, 2024). Ed.D bear significant responsibility in equipping practitioners to address emerging challenges in complex leadership roles (Walker and Haley-Mize, 2012). Since the 1990s, professional doctorates have rapidly proliferated in countries such as the United States, the United Kingdom, Australia, and New Zealand, becoming a prominent feature of the global graduate education landscape (Bourner et al., 2001; Kot and Hendel, 2012). Increasingly, countries worldwide are recognizing the value of professional doctorates in contributing to the knowledge economy, leading to the introduction and expansion of these degrees (Zambo et al., 2014). In China, with strong policy support, the Ed.D has experienced rapid growth in both enrollment and the number of institutions authorized to offer the degree.

Ed.D are essential in preparing educational leaders and scholars by equipping them with a strong theoretical foundation and robust research skills. However, Ed.D students face distinctive challenges in completing their doctoral studies (Geesa et al., 2020). Many Ed.D students pursue their studies part-time, balancing multiple life roles (Terry and Ghosh, 2015), leading to challenges in maintaining a work-life balance, managing role conflicts, and staying focused on dissertation work. At the same time, Ed.D students face lower graduation rates. For example, the postponement graduation rate of China’s education doctor is 79%, which is in a high state, far higher than the national 39.68% postponement graduation rate of doctoral students (Gao et al., 2020). It has been 15 years since China enrolled its first batch of Ed.D students in 2009. As of 2023, 8,467 students are enrolled, but only 1,148 students have successfully obtained their Ed.D degrees (China Education Professional Degree Graduate Education Network, 2024). Studies have shown that study time and academic self-efficacy are key factors influencing whether Ed.D students experience delays in graduation (Zhou et al., 2023; Yang et al., 2024). Ed.D students face dual pressures from work and family, which often result in low academic self-efficacy (Zhao and Sheng, 2022). Ed.D students’ research self-efficacy not only influences the degree of their effort, but also affects their learning motivation, attitudes, and engagement (Qin et al., 2022). Meanwhile, part-time doctoral students often experience distinct forms of anxiety arising from graduation requirements, career demands, and family responsibilities, accompanied by confusion and negative emotions, all of which may significantly impede their academic progress (Liu and Li, 2020; Bai et al., 2025). Some Ed.D students lack strong academic beliefs and exhibit low research self-efficacy, making them unable to cope with the significant academic challenges and pressures they encounter during their studies, which in turn leads to poor academic performance (Cai et al., 2020). The poor status of Ed.D students’ research self-efficacy and learning engagement is claimed to be the typical reason for their academic failure (Luo et al., 2023; Fredricks et al., 2004). It can be seen that many Ed.D students experience anxiety and self-doubt regarding their research abilities, which can lead to lower academic achievement. Moreover, the pressures of family and work make it difficult for them to ensure sufficient time for academic engagement, preventing them from achieving their academic achievement.

Academic achievement has long been a focal point of scholarly inquiry (Tao et al., 2022). While previous studies have highlighted the importance of self-efficacy as a key predictor of academic performance (Forester et al., 2004; Luo et al., 2023), limited research has specifically examined the role of research self-efficacy among Ed.D students, a group facing distinct academic and professional challenges (Kot and Hendel, 2012; Ari et al., 2022). Furthermore, learning engagement has been recognized as a crucial motivational factor influencing students’ academic outcomes (Fredricks et al., 2004; Cai et al., 2020; Hodgkin et al., 2024). However, the mechanisms through which research self-efficacy affects academic achievement, particularly via learning engagement, remain underexplored. Most existing studies has concentrated on undergraduate or Ph.D. populations (Han, 2024; Mackie and Bates, 2019; Willess, 2023; Wollast et al., 2023), or focused on variables such as master’s students’ self-regulation (Zhang et al., 2024). Despite the recognized importance of research self-efficacy, there is limited understanding of how to support students, particularly those in Ed.D programs, in developing their research capabilities and maintaining engagement with research activities (Kerrigan and Hayes, 2016; Jones, 2018). Therefore, this study aims to fill this gap by examining how research self-efficacy influences Ed.D students’ academic achievement, and whether learning engagement mediates this relationship. By doing so, the study contributes to a deeper understanding of how to support academic success in professional doctoral programs.

This study aims to explore the relationship between research self-efficacy and academic achievement among Ed.D students in China, with a particular focus on the mediating role of learning engagement. Given the significant impact of student engagement on both current performance and future success, it is crucial to understand how to effectively foster students learning engagement (Cents-Boonstra et al., 2021). Research training environment is one of the important antecedents of research self-efficacy (Livinƫi et al., 2021). Both contextual and individual factors significantly influence academic achievement (Owusu-Agyeman and Mugume, 2023; San and Guo, 2023). This study explores the factors influencing the academic achievement of Ed.D students, addressing a gap in the existing research. It also proposes pathways for universities to optimize Ed.D training programs and offers strategies for mentors to enhance students’ research self-efficacy and learning engagement. Therefore, the findings from this research will offer valuable insights for educators and academic institutions, guiding instructional strategies and professional development efforts aimed at supporting the academic development of Ed.D students.

## 2 Literature review

### 2.1 Social cognitive theory

This study is grounded in Albert Bandura’s Social Cognitive Theory (Bandura, 1986a), which introduces the concept of triadic reciprocal determinism. This model posits that human behavior is the result of the dynamic interplay among personal, environmental, and behavioral factors. One of the crucial personal factors that influences students’ engagement in tasks is self-efficacy. Self-efficacy, as a central tenet of Bandura’s theory, is defined as an individual’s belief in their ability to execute tasks successfully. This belief, in turn, significantly impacts their thoughts, motivation, and actions (Bandura, 1997). According to Bandura, self-efficacy represents an individual’s perception of their capacity to meet challenges and achieve desired outcomes. Research has demonstrated that higher self-efficacy is strongly associated with better performance, more positive attitudes, and higher academic success (Alhadabi and Karpinski, 2020). Self-efficacy beliefs are cultivated through four primary sources: actual performance accomplishments, vicarious experiences, social persuasion, and physiological states (Bandura, 1997). These sources influence how students approach tasks, regulate their behaviors, and persevere through challenges. Specifically, when learners feel capable of succeeding, they are more likely to engage effectively with their environment and adjust their actions to optimize learning opportunities (Schunk, 1995; Schunk and Pajares, 2009; Sökmen, 2021). The role of self-efficacy is further highlighted by its connection with emotional states. Positive emotions and moods tend to enhance self-efficacy, while negative emotions can detract from it, diminishing students’ ability to perform optimally (Robbins et al., 2004; Honicke et al., 2020; Vincent et al., 2021). A strong sense of self-efficacy is regarded as essential for graduate students’ research creativity and sustaining a successful and fulfilling teaching career (Han et al., 2024; Li et al., 2025; Täschner et al., 2025). Ed.D students with high self-efficacy, strong extrinsic motivation, and frequent communication with their mentors are more likely to graduate on time (Qin et al., 2022; Zhou et al., 2023). Bandura’s framework, therefore, provides a valuable lens for understanding how students with low self-efficacy may struggle with academic achievement, often due to their diminished belief in their own capabilities.

**Figure 1:**
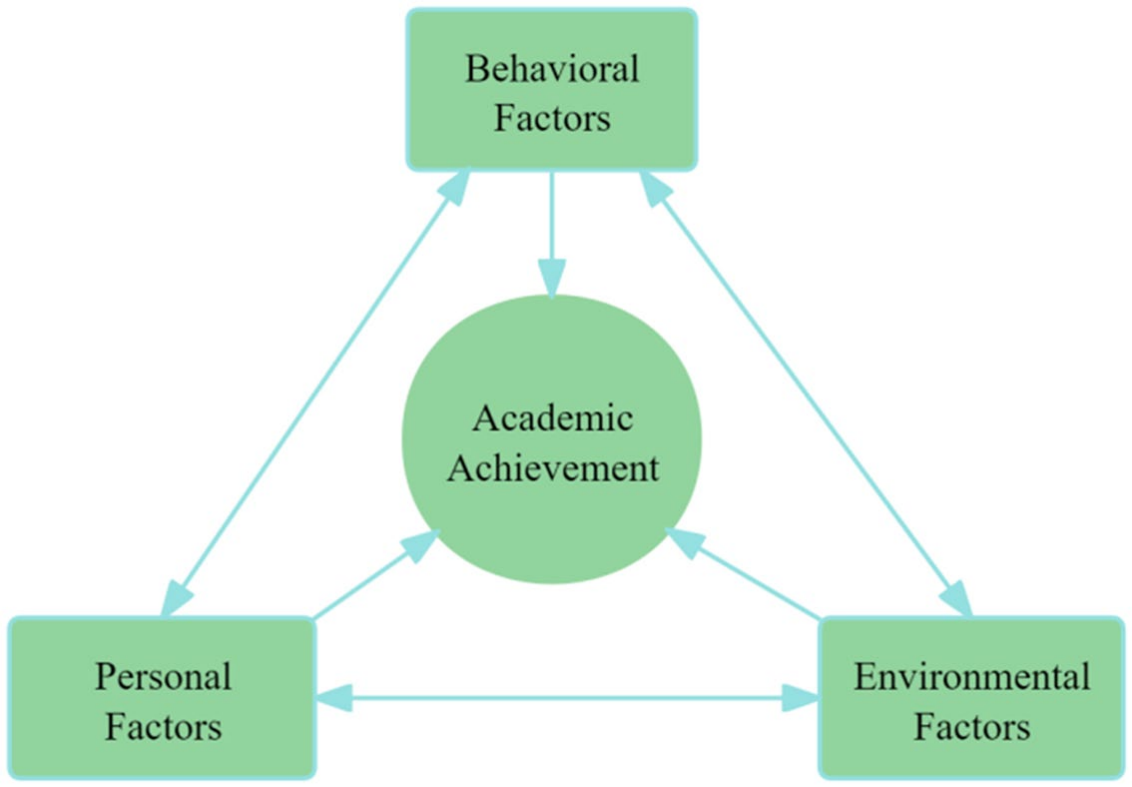
Triadic reciprocal factors on academic achievement.

According to Bandura’s model of dynamic reciprocal interaction, the academic achievement of Ed.D students is shaped by the continuous interplay among personal, behavioral, and environmental factors. As illustrated in Figure 1, this triadic model emphasizes that no single factor operates in isolation. In this study, personal factors are represented by research self-efficacy, referring to students’ beliefs in their ability to successfully engage in academic research. Behavioral factors are captured by learning engagement, which includes students’ cognitive effort, emotional involvement, and active participation in learning tasks. Environmental factors consist of institutional and mentor support, such as the quality of supervision, access to academic resources, and a supportive learning environment. These three elements, both independently and interactively, influence Ed.D students’ academic outcomes.

### 2.2 Research self-efficacy

Research self-efficacy, an adaptation of social cognitive theory, specifically addresses an individual’s belief in their ability to engage in research-related activities, including designing studies (Stadtlander et al., 2020). Research self-efficacy refers to a graduate student’s confidence in their ability to effectively define and conduct research (Elballah et al., 2024). Research self-efficacy has been identified as one of the strongest predictors of successful engagement in research activities (Livinƫi et al., 2021). Research self-efficacy concerns the belief in one’s ability to carry out tasks, such as conducting literature reviews, collecting and analyzing data, and writing reports or essays (Fokkens-Bruinsma et al., 2021). It is a key determinant of success in research, influencing both motivation and engagement in academic endeavors. Despite its importance, there is limited research exploring the interrelationships among the various factors that influence college students learning engagement through the lens of social cognitive theory (Shao and Kang, 2022).

Research self-efficacy is a key determinant of academic achievement in higher education. According to Bandura’s self-efficacy model, a student’s motivation and academic performance are shaped by their subjective perception of their ability to succeed (Bandura, 1986a, b). Research self-efficacy means students’ judgment of their ability to successfully perform academic tasks, which fosters positive attitude and confidence (Schunk, 1991; Schneider and Preckel, 2017). Students with high research self-efficacy are more likely to manage academic challenges, reflect on their learning experiences and adjust their academic behaviors to improve outcomes (Adams et al., 2020; Zysberg and Schwabsky, 2021). Higher self-efficacy can motivate students to set more challenging goals for themselves, creating a cycle of increased effort and success (Zimmerman, 1990). Specifically, students with greater research self-efficacy are more confident in their ability to complete research tasks (Honicke et al., 2023), while those with lower self-efficacy may avoid challenges and underperform in research. Based on these findings, we propose the following hypothesis:

*H1*: Research self-efficacy is a positive predictor of academic achievement among Ed.D students.

### 2.3 Learning engagement

Learning engagement has multifaceted definitions. While the term may appear intuitive and straightforward, a closer examination reveals a wide range of interpretations. Natriello (1984) defines engagement as the extent to which students are involved in the activities provided through the school program. Kuh (1991) formally introduced the concept of student engagement in higher education, emphasizing its role in fostering student learning and development. Schaufeli et al. (2002) further refine this definition, emphasizing engagement as a work-focused, positive psychological state. Skinner et al. (2009) view engagement as the behavioral manifestation of motivation, reflected in students’ active participation in academic activities. More recently, Wong and Liem (2022) define learning engagement as a psychological state in which students feel activated, exert effort, and become deeply absorbed in learning tasks. Some researchers define learning engagement as the level of attention, curiosity, interest, optimism, and enthusiasm students exhibit in the learning process, which drives academic success (Bakker et al., 2015; Gan et al., 2024). Although scholars define learning engagement from different theoretical perspectives, there is broad consensus that learning engagement represents a multidimensional psychological construct encompassing affective and cognitive components such as interest, effort, and absorption.

Fredricks et al. (2004) proposed that learning engagement encompasses three dimensions: behavioral engagement, cognitive engagement, and emotional engagement. Behavioral engagement includes participation in academic and class-related activities, attention, engagement, concentration, completing assignments, and adherence to class rules. Emotional engagement refers to students’ positive feelings toward teachers, classmates, class activities, interests, and their identification with the school or subject area. Cognitive engagement involves commitment to learning, self-regulation, persistence, and the effort to understand complex ideas or master difficult skills (Fredricks et al., 2016). Over time, scholars have increasingly accepted this three-dimensional framework. Based on existing research, this study defines learning engagement as the degree of effort, persistence, concentration, problem-solving, and emotional involvement that a student demonstrates during learning activities, encompassing cognitive, emotional, and behavioral dimensions.

Research self-efficacy has been found to be a strong predictor of learning engagement (Sökmen, 2021). Existing research has consistently shown that research self-efficacy is positively related to learning engagement (Sanchez-Cardona et al., 2012). Findings also suggest that research self-efficacy enhances students’ engagement in learning (Azila-Gbettor et al., 2021). Students who possess high self-efficacy tend to exhibit higher levels of behavioral, motivational, and cognitive engagement compared to their peers (Akpan and Umobong, 2013). In contrast, students with low self-efficacy often set lower learning goals, have negative attitudes toward academic challenges, and struggle to employ effective learning strategies (Fan and Williams, 2010; Froiland and Worrell, 2016). Based on these findings, we propose the following hypothesis:

*H2*: There is a positive association between Ed.D students’ research self-efficacy and learning engagement.

### 2.4 Academic achievement

Achievement is an important concept, signifying a positive outcome such as the attainment of set goals, the realization of aspirations, and the transformation of thoughts into actions (Erdoğdu, 2019). Academic achievement refers to the performance outcomes that reflect the extent to which individuals have accomplished specific objectives within educational settings, such as schools, colleges, and universities (Steinmayr et al., 2014). Previous studies have highlighted the crucial role of learning engagement in enhancing students’ academic success, facilitating both knowledge acquisition and academic performance (Bresó et al., 2011; Shao and Kang, 2022). Specifically, learning engagement is a strong predictor of academic performance, with students who actively engage in tasks—such as concentrating during lessons and completing class assignments—generally achieving better outcomes than those who struggle to manage these activities (Northey et al., 2018; Klapp et al., 2024). However, despite these findings, the literature reveals a gap in identifying clear and implementable methods for fostering and sustaining student engagement.

Research has consistently demonstrated the significant role of research self-efficacy, learning engagement, and achievement in an academic setting. Learning engagement has been found to significantly mediate the relationship between psychological characteristics and academic achievement across diverse student populations (Wang and Yan, 2019). Furthermore, both student engagement have been identified as a key mediator in the influence of teacher support on students’ academic performance (Huang and Wang, 2023). Specifically, engagement has been shown to mediate the direct influence of student motivation on academic outcomes. Building on these findings, we hypothesize the following:

*H3*: There is a positive association between Ed.D students’ learning engagement and academic achievement.

*H4*: Learning engagement is expected to positively mediate the relationship between research self-efficacy and academic achievement among Ed.D students.

### 2.5 The present study

**Figure 2:**
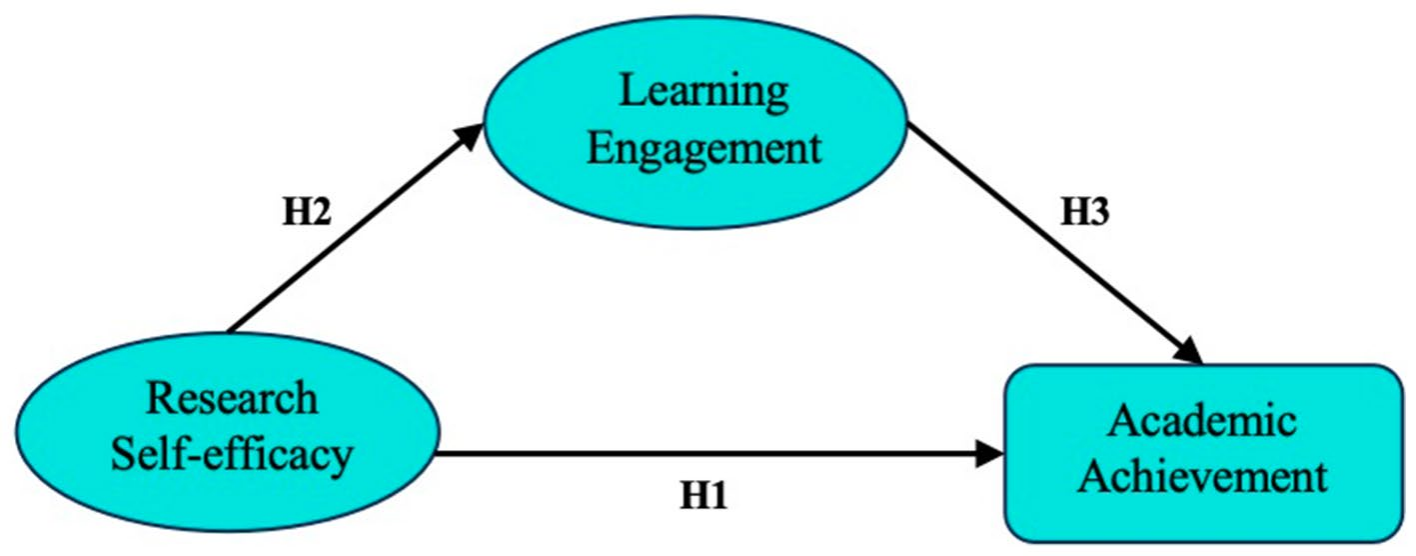
Conceptual framework.

This study aims to provide a comprehensive understanding of the relationship between research self-efficacy and academic achievement among Ed.D students, as illustrated in Figure 2. In addition, learning engagement was tested as a potential mediator to explore how research self-efficacy impacts academic achievement. To assess the relationships and mediating effects among academic self-efficacy, learning engagement, and academic achievement, both correlation and mediation analyses were conducted.

## 3 Methods

### 3.1 Participants

The participants in this study were recruited from Beijing Normal University and Shaanxi Normal University, both of which are directly administered by the Ministry of Education of the People’s Republic of China. These two universities were among the first institutions in China to establish Ed.D programs, positioning them as pioneers and models in the field of professional doctoral education (Qin and Song, 2021). With extensive experience in cultivating Ed.D students, these universities have developed mature training systems. The establishment of Ed.D programs in China began in 2010, and by 2022, a total of 31 universities across the country had been authorized to admit and cultivate Ed.D students. Among these institutions, Beijing Normal University and Shaanxi Normal University are the two universities that admit the largest number of Ed.D students (Hu and Zhou, 2023). In recent years, each of these two institutions has admitted over 100 Ed.D students annually. They enroll students from diverse regions across the country, reflecting significant geographical and cultural diversity, which enhances the representativeness and generalizability of the study findings. These two normal universities, located in eastern and western China respectively, allow the study to account for regional differences. This study focused on these two institutions due to their early adoption of Ed.D programs, large enrollment of Ed.D students, and mature administrative structures for professional education, which facilitated access to a sufficient and relevant sample.

A total of 351 Ed.D students were invited to participate, and after excluding invalid responses, a final sample of 310 valid responses was retained. The survey was disseminated through collaboration between university faculty and student mentors, who shared the survey link across over 10 Ed.D student groups via WeChat. Before the questionnaire was distributed, participants received written information outlining the study’s objectives and procedures. They were also informed that their participation was entirely voluntary and that their responses would remain anonymous. Data collection was facilitated through the Questionnaire Star platform, and ethical approval for the study was obtained prior to the commencement of data gathering. As detailed in Table 1, the final sample consisted of 143 males (46.13%) and 167 females (53.87%), with an average age of approximately 37 years. All participants voluntarily agreed to participate in the study.

**Table 1:** Participant profile.

Items	Variables	n	%
College	Beijing Normal University	158	50.97
	Shaanxi Normal University	152	49.03
Gender	Male	143	46.13
	Female	167	53.87
Age	less than 30	35	11.29
	30–35	99	31.93
	36–40	80	25.81
	Greater than 41	96	30.97
Grades	First year	45	14.52
	Second year	90	29.03
	Third year	116	37.42
	Third year	59	19.03

### 3.2 Measures

To ensure the reliability and validity of the measurement tools, we utilized well-established scales widely recognized in academic research, that is the research self-efficacy scale developed by Elballah et al. (2024), the learning engagement scale by Fredricks et al. (2016), and the academic achievement scale by Tao et al. (2022). All scales underwent translation and back-translation procedures to ensure linguistic accuracy and cultural appropriateness, followed by expert consultations for optimization. Based on relevant literature, this study sorted out the main variables and relevant questionnaires of research self-efficacy, learning engagement and academic achievement. In combination with the learning situation of Chinese Ed.D students, this study designed the relevant questionnaire survey. The reliability and validity of the questionnaire were tested by SPSS22.0 and Amos23.0 to ensure the validity and reliability of the survey.

The reliability of the formal questionnaire was assessed using SPSS 22.0 software, and the results are presented in Table 2. The overall reliability coefficients for the Academic Achievement Scale, Research Self-Efficacy Scale, and Learning Engagement Scale all exceeded 0.9. Additionally, the reliability values for the sub-dimensions of each scale were all greater than 0.8. These findings indicate that the formal questionnaire demonstrates a high level of reliability.

**Table 2:** Results of the scales reliability analysis.

Scales	Dimensionalities	Number	Cronbach’s alpha	
The research self-efficacy scale	Curriculum learning	5	0.960	0.963
	Research activity	9	0.953	
	Teaching practice	4	0.951	
The learning engagement scale	Behavioral engagement	6	0.949	0.968
	Cognitive engagement	5	0.965	
	Emotional engagement	6	0.933	0.944
The academic achievement scale	Research achievements	7	0.908	
	Knowledge accumulation	6	0.890	

The research self-efficacy scale: The research self-efficacy scale consists of 18 items, divided into three sub-scales: the curriculum learning scale (5 items, e.g., “I can complete the learning tasks of professional courses very well”), the research activities scale (9 items, e.g., “I have the ability to perform scientific research tasks well”), and the teaching practice scale (4 items, e.g., “I can apply the knowledge and skills I have learned to social practice”). Participants responded to these items using a 5-point Likert scale, ranging from 1 (strongly disagree) to 5 (strongly agree), with higher scores indicating greater research self-efficacy. The Average Variance Extracted (AVE) for the three sub-scales were as follows: 0.68 for the curriculum learning sub-scale, 0.63 for the research activities sub-scale, and 0.65 for the social practice self-efficacy sub-scale. These values exceed the recommended thresholds of 0.50 for AVE and 0.70 for CR, indicating strong convergent validity and internal consistency (Abdollahi and Noltemeyer, 2018).

The learning engagement scale: The scale comprises 17 items, organized into three sub-scales: the behavioral engagement scale, cognitive engagement scale and emotional engagement scale. Sample items include: “I often read literature related to my major and field” “I can concentrate on the learning process” and “I am confident I will complete my studies successfully.” Responses are recorded on a 5-point Likert scale, ranging from 1 (strongly disagree) to 5 (strongly agree), with higher scores indicating greater learning engagement. The confirmatory factor analysis (CFA) further supported the model fit, with indices as follows: X^2^/df = 2.627, RMSEA = 0.036, GFI = 0.971, TLI = 0.982, CFI = 0.995, and SRMR = 0.018, indicating good structural validity. Factor loadings ranged from 0.451 to 0.736.

The academic achievement scale: The academic achievement scale consists of 13 items across two dimensions: research ability achievement and knowledge accumulation. Representative items include “Master the scientific research methods” and “Understand basic theoretical knowledge.” Responses are rated on a 5-point scale, with higher scores indicating greater academic achievement. The confirmatory factor analysis (CFA) confirmed the one-dimensional structure of the scale, with good model fit: X^2^/df = 2.502, RMSEA = 0.036, GFI = 0.972, TLI = 0.978, CFI = 0.981, and SRMR = 0.025, supporting its structural validity. Factor loadings ranged from 0.539 to 0.698. This scale has also been widely used in prior research and has been shown to have strong reliability and validity.

### 3.3 Data analysis

Data were processed and analyzed using SPSS 22.0 and Amos 23.0 software. These software packages were selected due to their widespread application in social science research and their strong capabilities in conducting structural equation modeling (SEM), reliability analysis, and mediation testing (Feng, 2015; Wu, 2018). These software tools are widely used and recognized for their reliability in social science research, particularly in conducting descriptive statistics, reliability analysis, and structural equation modeling. The analytical process involved several steps: (1) Preliminary Screening: Collected data were first screened for validity. Questionnaires were excluded from the analysis if they demonstrated unusually short completion times, exhibited logical inconsistencies, or showed a response pattern of selecting the same option across all items. (2) Descriptive Statistics and Correlation Analysis: Descriptive statistics were calculated to summarize the sample characteristics. Pearson correlation analysis was conducted to explore relationships among the study variables. In this study, 310 valid questionnaires were collected to verify the hypothesis proposed above. The statistical software SPSS22.0 was used to analyze the scales of research self-efficacy, learning engagement and academic achievement. (3) Differences in demographic variables among variables. This study examined the differences between demographic variables and research self-efficacy, learning engagement and academic achievement. After the independent sample *T*-test for gender, it was found that there was no significant difference between male and female students in research self-efficacy, learning engagement and academic achievement of Ed.D students. The specific results are shown in the Table 3.

**Table 3:** Independent sample *T*-test for gender.

Variables	Gender	Number	Mean value	*F*-value	*T*-value	Significance
Research self-efficacy	Male	143	3.716	0.643	−0.782	0.433
	Female	167	3.632			
Learning engagement	Male	143	3.518	1.851	−0.193	0.837
	Female	167	3.533			
Academic achievement	Male	143	3.597	0.618	0.781	0.435
	Female	167	3.515			

(4) Exploratory Factor Analysis (EFA): To assess the construct validity of the measurement instruments, exploratory factor analysis (EFA) was first conducted using SPSS 22.0. The Kaiser-Meyer-Olkin (KMO) values for the Research Self-Efficacy Scale (0.931), Learning Engagement Scale (0.952), and Academic Achievement Scale (0.923) indicated excellent sampling adequacy. Bartlett’s Test of Sphericity was significant for all scales (*p* < 0.001), confirming that the data were suitable for factor analysis.

(5) Confirmatory Factor Analysis (CFA): Confirmatory factor analysis was performed using Amos 24.0 to assess the discriminant validity of the main variables, which included research self-efficacy, learning engagement and academic achievement. According to Wu (2010), when x^2^/df is less than 5, the model fit is acceptable, and when its value is less than 3, the model fit is good. When the approximate error of root mean square (RMSEA) is <0.05, the model has a good fit. When 0.05 < RMSEA<0.08, the model fit was acceptable. When the normalized residual root mean square (SRMR) is less than 0.05, the model has a good fitting effect. The goodness of fit index (GFI) is greater than 0.9, indicating that the model has a good fitting effect. The fitting criteria of comparative fitting index (CFI) and non-standard fitting index (TLI) are all above 0.90. When CFI and TLI are all above 0.95, it indicates that the model fitting effect is excellent. This model outperformed other competing models, thereby indicating robust discriminant validity among the five principal variables under investigation.

## 4 Results

### 4.1 Descriptive statistics and correlation analysis

To examine the relationships among research self-efficacy, learning engagement, and academic achievement, Pearson correlation coefficients were computed. The mean score for research self-efficacy was 3.42 (SD = 1.08), indicating a moderate level of self-efficacy. In comparison, the mean score for learning engagement was slightly higher at 3.39 (SD = 0.95), also reflecting a moderate level. The mean score for academic achievement was 3.41(SD = 1.09), indicating a moderate level of academic achievement. Descriptive statistics and correlation coefficients are presented in Table 4. The findings revealed significant associations between research self-efficacy, learning engagement, and academic achievement. Specifically, the correlation coefficients were 0.87 between research self-efficacy and learning engagement, 0.81 between research self-efficacy and academic achievement, 0.89 between learning engagement and academic achievement.

**Table 4:** Correlation analyses.

Variables	*M*	SD	1	2	3	4	5	6	7	8	9
Predictor variables											
1. Research self-efficacy	3.42	1.08	1								
2. Curriculum learning	3.32	1.13	0.863**	1							
3. Research activities	3.43	1.05	0.892**	0.941**	1						
4. Teaching practice	3.52	1.06	0.892**	0.832**	0.811	1					
Mediator variables											
5. Learning Engagement	3.39	0.95	0.873**	0.902**	0.916**	0.901**	1				
6. Behavioral Engagement	3.36	1.03	0.821**	0.871**	0.896**	0.962**	0.861**	1			
7. Cognitive Engagement	3.41	0.87	0.789**	0.829**	0.845**	0.959**	0.863**	0.811**	1		
8. Emotional Engagement	3.40	0.96	0.827**	0.853**	0.887**	0.955**	0.865**	0.872**	0.809**	1	
Target variable											
9. Academic Achievement	3.41	1.09	0.808**	0.887**	0.841**	0.812**	0.753**	0.735**	0.779**	0.834**	1

**p < 0.01.

The results demonstrated significant associations among the three variables (Table 4). Correlation analysis indicated that the three dimensions of research self-efficacy—curriculum learning self-efficacy, research activities self-efficacy and teaching practice self-efficacy—were positively related to academic achievement, as were the three dimensions of learning engagement—behavioral engagement, cognitive engagement and emotional engagement. Moreover, each dimension of research self-efficacy was significantly and positively associated with all dimensions of learning engagement (*p* < 0.01). These findings highlight the potential mediating role of learning engagement in the relationship between research self-efficacy and academic achievement, providing a basis for further investigation into this intermediary effect.

### 4.2 Hypothesis testing

Drawing from the existing literature, several hypotheses were formulated to address the research questions. A hypothesis is deemed significant if its t-value surpasses 1.96 (Sarstedt et al., 2021). Hypotheses were examined using Amos 23.0 software, employing bootstrap samples to contemporary standards to scrutinize the mediating roles of various constructs. The model included direct paths from mentor support to research self-efficacy, scientific engagement, and research creativity. Indirect effects were examined using the bootstrap method to estimate confidence intervals. The outcomes of these analyses are presented in Table 5.

**Table 5:** Structural relationships and hypothesis testing.

Hypothesis	Path	Path coefficient	*t* statistics	*p*-values	Decision
H1	Research self-efficacy → Academic achievement	0.332	10.986	<0.01	Supported
H2	Research self-efficacy → Learning engagement	0.526	17.816	<0.01	Supported
H3	Learning engagement → Academic achievement	0.387	10.398	<0.01	Supported

The model in this study is the simplest mediation model, involving three latent variables and only one mediation variable. Therefore, Process V4.0 was used to test the mediation effect. The test results are shown in the table below. According to Table 6, the research self-efficacy of Ed.D students can significantly predict academic achievement (*β* = 0.796, *p* < 0.001). When the intermediary variable of learning engagement is included, the direct prediction effect of academic self-efficacy is weakened (*β* = 0.439, *p* < 0.001). The research self-efficacy of Ed.D students can significantly predict their learning engagement (*β* = 0.821, *p* < 0.001), and learning engagement can also significantly predict their academic achievement (*β* = 0.432, *p* < 0.001). In addition, according to the test results in Table 7, the total effect value is 0.848, and the confidence interval does not include 0, which indicates that the research self-efficacy of Ed.D students will have an impact on academic achievement in general. Meanwhile, the direct effect value is 0.471, the BootLLCI value of the direct effect is 0.368, and the BootULCI value is 0.573. The 95% confidence interval of the direct effect Bootstrap does not include 0. This indicates that the self-efficacy of Ed.D students has a direct impact on academic achievement. Lastly, the mediation effect value was 0.378, the BootLLCI value of the mediation effect was 0.291, and the BootULCI value was 0.467. It can be seen that the confidence interval does not include 0, which indicates that the mediation effect is significant. In addition, the direct effect accounted for 55.43%, and the intermediate effect accounted for 44.57%, and both were significant. The results show that the involvement of Ed.D students plays a mediating role between academic self-efficacy and academic achievement. Therefore, the research hypothesis H4 is verified.

**Table 6:** Testing the mediation model of learning engagement.

Predictor		Academic achievement		Learning engagement		Academic achievement	
		*β*	*t*	*β*	*t*	*β*	*t*
Result variables	Research Self-efficacy	0.796	31.187***	0.821	34.160***	0.439	10.813***
	Learning engagement					0.432	10.593***
	*R* square	0.632		0.673		0.693	

All variables in the model are substituted into the regression equation after standardization, and *** indicates *P* < 0.001.

**Table 7:** Decomposition of each effect.

Effect type	Effect	BootSE	BootLLCI	BootULCI	Effect ratio
Mediating effect	0.378	0.045	0.291	0.467	44.57%
Direct effect	0.471	0.052	0.368	0.573	55.43%
Total effect	0.848	0.027	0.794	0.899	

**Figure 3:**
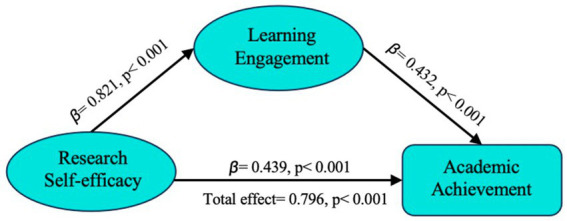
Mediating effect model.

To sum up, Ed.D students’ research self-efficacy can directly affect their academic achievement, and can also indirectly affect their academic achievement through the intermediary variable of learning engagement. The path analysis diagram among the three variables is shown in Figure 3. In Figure 3, the independent variable is research self-efficacy, the mediating variable is learning engagement, and the dependent variable is academic achievement. This model suggests that research self-efficacy influences academic achievement both directly and through learning engagement. Specifically, the research results show that if the Ed.D students have a strong sense of research self-efficacy, they are more likely to invest more time and energy in study and research, and obtain higher academic achievement through sustained engagement and perseverance.

## 5 Discussion

This study examines the relationship between research self-efficacy and academic achievement, with a focus on the mediating role of learning engagement among Ed.D students. The correlation analysis revealed significant positive associations between research self-efficacy, learning engagement, and academic achievement. Further mediation analysis demonstrated that research self-efficacy not only has a direct effect on academic achievement but also exerts an indirect effect through learning engagement. These findings provide support for Hypotheses 1, 2, 3, and 4.

First, research self-efficacy was found to be a direct and significant predictor of academic achievement among Ed.D students, supporting Hypothesis 1. The study confirmed that research self-efficacy is a significant predictor of academic achievement among Ed.D students. This finding aligns with previous studies (Nyunt et al., 2023; Qin et al., 2022; Zhou et al., 2023). According to social cognitive theory, self-efficacy reflects an individual’s confidence in their ability to perform specific tasks, which in turn influences their actions, attitudes, and performance (Bandura, 1982). Doctoral students who feel confident that they can successfully perform research tasks are more interested and motivated to conduct research (Bishop and Bieschke, 1998; Du et al., 2020). The study also explored the three dimensions of the research self-efficacy scale for Ed.D students and found that they reported relatively high levels of self-efficacy in curriculum learning and teaching practice. However, their self-efficacy in research activities was comparatively lower. Over 100 students were uncertain about their ability to publish articles that meet graduation requirements. This suggests that, while Ed.D students possess substantial work experience and confidence in their teaching abilities, their research skills require further development. Research self-efficacy reflects an individual’s belief in their capacity to perform specific academic tasks, and this belief significantly shapes their actions, attitudes, and overall performance (Jones et al., 2024). A strong sense of self-efficacy is a critical factor in promoting teachers’ development (Täschner et al., 2025). Greater self-efficacy promotes further skill development and leads to more successful performance (Overall et al., 2011; Vizoso et al., 2019). Specifically, students who report higher levels of research self-efficacy are more proactive in embracing academic challenges, exhibiting greater perseverance and effort in their academic pursuits. A lack of research self-efficacy can result in diminished confidence when students encounter research-related challenges, potentially hindering their academic progress. Therefore, we should pay more attention to the cultivation of Ed.D students’ research self-efficacy.

Second, the study supports Hypothesis 2 by demonstrating that research self-efficacy positively predicts learning engagement among Ed.D students. This finding is consistent with previous research highlighting the significant relationship between self-efficacy and student engagement (Bresó et al., 2011; Olivier et al., 2019; Luo et al., 2023). The study further explored the factors influencing Ed.D students’ learning engagement, revealing that many students face the challenge of balancing their academic and professional responsibilities. These struggles can negatively impact associated outcomes, including research productivity and long-term academic success (Litson et al., 2021). Ed.D students’ research self-efficacy not only influences the level of their goals and the degree of effort they exert, but also affects their learning motivation, learning attitude, and learning engagement (Qin et al., 2022). Ed.D students, in particular, often encounter barriers to developing or maintaining high research self-efficacy due to competing work and study demands. Strong self-efficacy beliefs empower individuals to tackle challenging tasks, while those with lower self-efficacy may struggle to persist and often abandon projects when faced with difficulties (Jonathans et al., 2024). Therefore, fostering a stronger sense of research self-efficacy serve as a critical step toward enhancing students’ engagement with their academic pursuits.

Third, the findings underscore that students’ learning engagement has a significant positive influence on academic achievement, thereby supporting Hypothesis 3. This result aligns with prior research demonstrating that learning engagement is a strong predictor of academic performance (Northey et al., 2018; Sanchez-Cardona et al., 2012; Sökmen, 2021). Learning engagement is a key factor in enhancing the academic achievement of Ed.D students (Jian, 2022; Zhou et al., 2023; Qin et al., 2025). Although the reasons for poor academic achievement are complex, the most prominent factor is Ed.D students’ insufficient study time. If Ed.D students devote more than 3.5 h per day to their studies on average, the risk of delayed graduation can be effectively mitigated (Yang et al., 2024). It can be seen that achieving academic success and meeting graduation standards require a significant amount of time and effort. Obviously, sustained engagement is closely linked to higher academic success. This study highlights the role of Ed.D students’ learning engagement in achieving academic achievement.

Finally, the study also reveals that learning engagement plays a critical mediating role between research self-efficacy and academic achievement among Ed.D students, supporting Hypothesis 4. Specifically, Ed.D students’ research self-efficacy positively influences academic performance through the mediation of learning engagement. This is consistent with the previous studies stressing learning engagement plays a mediating role between academic motivation and academic achievement (Alemayehu and Chen, 2023; Fong et al., 2023; Han et al., 2024; Qin et al., 2025). This study suggests that Ed.D students who perceive themselves as capable of conducting research are more likely to invest cognitively, emotionally, and behaviorally in their academic activities, which in turn enhances their academic performance. On the contrary, Ed.D students with low research self-efficacy often exhibit reduced motivation, limited persistence, and passive engagement in learning tasks. They may experience heightened anxiety and self-doubt when confronted with research challenges, which negatively affects their ability to complete academic work efficiently. As research has shown, research self-efficacy directly promotes self-monitoring and engagement in the learning process, and facilitates progress in research activities (Alemayehu and Chen, 2023; Fong et al., 2023). High research self-efficacy is connected to the attitudes and behaviors of researchers and ultimately with their research performance (Feldman and Kubota, 2015; Livinƫi et al., 2021). Higher levels of Ed.D students’ research self-efficacy not only enhance student engagement but also lead to better academic outcomes. The mediating effect suggests that Ed.D students’ research self-efficacy does not directly influence achievement in isolation, but rather exerts its effect through students’ sustained efforts, concentration, emotional investment, and problem-solving during the learning process.

## 6 Implications

The findings of this study demonstrate significant positive associations between research self-efficacy, learning engagement, and academic achievement among Ed.D students. Based on these results, the study offers theoretical and practical implications aimed at improving Ed.D students’ academic achievement. These suggestions focus on three key areas: universities, educators, and the students themselves, with the ultimate goal of fostering enhanced research skills and academic success.

### 6.1 Theoretical implications

The findings of this study make several theoretical contributions to the understanding of Ed.D programs. On the one hand, this study reinforces Bandura’s (1997) self-efficacy theory, which posits that individuals’ beliefs in their capabilities influence their motivation, learning behaviors, and achievement outcomes. While the self-efficacy framework has been widely applied in undergraduate and general graduate education contexts (Mackie and Bates, 2019; Wollast et al., 2023; Zhang et al., 2024), it has received relatively limited attention within the domain of professional doctoral education, particularly Ed.D programs (Willess, 2023). This study bridges that gap by demonstrating that Ed.D students with higher levels of research self-efficacy are more engaged in learning and, consequently, more likely to achieve academic success. On the other hand, this study contributes to the theoretical development of learning engagement as a mediating variable. Previous research has acknowledged the importance of engagement as a multidimensional construct—behavioral, emotional, and cognitive—that links motivation to academic performance (Fredricks et al., 2004; Appleton et al., 2006). However, within the context of Ed.D students, engagement is often under-theorized and seldom conceptualized as a mediating process linking psychological traits to measurable academic outcomes. This study not only advances the theoretical model of student engagement within Ed.D programs but also underscores the importance of fostering engagement as a means to enhance academic success.

### 6.2 Practical implications

This study also offers practical implications by empirically validating the significant relationships among research self-efficacy, learning engagement, and academic achievement in Ed.D students.

Firstly, the findings of this study provide valuable insights for university administrators. Universities should offer more resources and learning opportunities to support Ed.D students, enhance their sense of competence in research, and help them build self-confidence. There needs to be a closer link between universities and relevant departments to better support Ed.D students (Hodgkin et al., 2024). For example, Northeast Normal University in China has implemented the University–Government–School (UGS) model, establishing the L–3R (leadership, research, reflection, and renewal) competency framework for Ed.D training (Wu et al., 2022). Such cooperation between universities and Ed.D students’ workplaces can help alleviate workload pressures and provide stronger research support. There is also practitioner-focused doctoral programs, findings point to equipping doctoral students with skills and knowledge in educational research (Ari et al., 2022), offering students sufficient opportunities to achieve their development. Meanwhile, universities should align their offerings with the needs and expectations of their students, designing targeted courses and initiatives that foster active engagement in learning. The Ed.D program is specifically designed for experienced practitioners and aims to deepen their theoretical understanding to inform educational practice. Its curriculum typically includes general, specialized, and practical courses. Activities such as academic exchanges, field investigations, and thematic seminars promote the integration of theory and practice, enhancing students’ learning motivation and efficiency, and helping them successfully complete their degrees. Universities can organize research seminars and invite experts to give lectures to further stimulate student interest and participation in research. Students’ engagement and participation are influenced by the degree to which the university environment promotes their sense of autonomy and relatedness. These perceptions, in turn, significantly affect their self-efficacy and academic performance (Pajares and Schunk, 2001). Therefore, universities should prioritize creating a positive research atmosphere and developing platforms for academic exchange to strengthen Ed.D students’ research self-efficacy. In addition, Ed.D students often face dual pressures from work and family, which can lead to low academic self-efficacy and a perceived lack of research competence (Zhao and Sheng, 2022; Bai et al., 2025). Providing Ed.D students with more flexible and diverse learning format—such as combining regular online learning with intensive face-to-face instruction during holidays—can ensure sufficient study time while also enhancing the quality of teaching.

Secondly, mentors serve as guides, facilitators, and supporters throughout Ed.D students’ pursuit of their doctoral degrees. Their role spans the entire learning journey, playing a crucial part in various stages such as identifying research problems, learning research methodologies, and selecting topics for academic papers (Guo et al., 2020). The degree to which mentors encouraged students to think and act autonomously predicted greater research self-efficacy (Overall et al., 2011; Livinƫi et al., 2021). Students are more productive, and their engagement and psychological resources are increased under supportive supervision, which ultimately significantly increases their research productivity (Khuram et al., 2023). Providing constructive feedback and encouraging goal setting helps foster students’ confidence and competence in their academic pursuits (Zimmerman, 2000; Martínez-López et al., 2024). Therefore, mentors establishing a collaborative learning community for Ed.D students can be highly beneficial. Within this community, mentors and students work together to explore research designs and carry out dissertation projects. Such a learning community fosters mutual support and collaborative interaction among students, enhances learning efficiency, and increases effective study time. Activities such as academic writing, involvement in research projects, and participation in reading groups not only reinforce consistent learning but also enhance students’ academic competence and the overall quality of mentor–student relationships.

Finally, Ed.D students must continuously engage in effective learning strategies to enhance their research capabilities. Balancing the demands of work and learning often exposes doctors to various challenges (Van Doorsselaere, 2024). To overcome these difficulties, Ed.D students need to cultivate qualities such as diligence, dedication, and resilience, embracing the research process as an opportunity for growth. Ed.D students should enhance their awareness of time management, strengthen their time management skills, and effectively balance their study and work schedules. During their academic journey, they should identify personalized methods to address the obstacles they encounter in both research and learning. Research indicates that higher levels of student engagement are directly associated with improved academic achievement (Zeng et al., 2023). Students who were more confident in their academic skills tended to regulate their effort and manage their study time and environment more effectively than students lower in self-efficacy (Van Rooij et al., 2018). Ed.D students, therefore, should establish clear research goals, maintain open communication with their mentors, and seek academic guidance to ensure consistent progress. When faced with difficulties, students should seek support from instructors, peers, or mentors, and to develop effective emotional regulation strategies to maintain a positive mindset. This not only boosts their academic performance but also enhances their research self-efficacy, leading to a more confident and proactive approach to their academic research.

## 7 Limitations

This study demonstrated the pathways through which research self-efficacy influences the academic achievement of Ed.D students, offering valuable insights for fostering positive psychological development and enhancing academic achievement. Despite these contributions, there are some limitations to consider. First, the study focused exclusively on the impact of research self-efficacy on academic achievement. To provide a more comprehensive understanding, subsequent research should incorporate additional factors, such as academic atmosphere, teacher guidance that may further illuminate the mechanisms underlying academic success. Secondly, this study examined learning engagement as a mediator, but future investigations could explore the potential mediating roles of other variables, such as self-esteem, goal orientation, and self-regulation, which may also contribute to academic achievement.

## 8 Conclusion

This study explores the relationship between research self-efficacy and academic achievement, with a particular focus on the mediating role of learning engagement. The findings indicate that research self-efficacy, learning engagement, and academic achievement are positively correlated. Furthermore, the study confirms that research self-efficacy enhances academic achievement by fostering greater learning engagement. This research contributes to the expanding body of knowledge on learning engagement and provides valuable insights for designing interventions aimed at improving Ed.D students’ academic achievement. Overall, the findings offer both theoretical and practical guidance for university administrators and students, broadening the understanding of how research self-efficacy, learning engagement, and academic achievement are interconnected.

## Data Availability

The original contributions presented in the study are included in the article/Supplementary material, further inquiries can be directed to the corresponding author.

## References

[ref1] Abdollahi A. Noltemeyer A. (2018). Academic hardiness: mediator between sense of belonging to school and academic achievement? J. Educ. Res. 111, 345–351. doi: 10.1080/00220671.2016.1261075

[ref2] Adams A. M. Wilson H. Money J. Palmer-Conn S. Fearn J. (2020). Student engagement with feedback and attainment: the role of academic self-efficacy. Assess. Eval. High. Educ. 45, 317–329. doi: 10.1080/02602938.2019.1640184

[ref3] Akpan I. D. Umobong M. E. (2013). Analysis of achievement motivation and academic engagement of students in the Nigerian classroom. AJIS 2, 385–390. doi: 10.5901/ajis.2013.v2n3p385

[ref4] Alemayehu L. Chen H. L. (2023). The influence of motivation on learning engagement: the mediating role of learning self-efficacy and self-monitoring in online learning environments. Interact. Learn. Environ. 31, 4605–4618. doi: 10.1080/10494820.2021.1977962

[ref5] Alhadabi A. Karpinski A. C. (2020). Grit, self-efficacy, achievement orientation goals, and academic performance in university students. Int. J. Adolesc. Youth 25, 519–535. doi: 10.1080/02673843.2019.1679202

[ref6] Appleton J. J. Christenson S. L. Kim D. Reschly A. L. (2006). Measuring cognitive and psychological engagement: validation of the student engagement instrument. J. Sch. Psychol. 44, 427–445. doi: 10.1016/j.jsp.2006.04.002

[ref7] Ari F. Vasconcelos L. Tang H. Grant M. M. Arslan-Ari I. Moore A. L. (2022). Program evaluation of an online EdD. in learning design and technologies: Recent graduates’ perspectives. Tech Trends 66, 699–709. doi: 10.1007/s11528-022-00744-7, PMID: 40381027

[ref8] Azila-Gbettor E. M. Mensah C. Abiemo M. K. Bokor M. (2021). Predicting student engagement from self-efficacy and autonomous motivation: a cross-sectional study. Cogent Educ. 8:1942638. doi: 10.1080/2331186X.2021.1942638

[ref9] Bai F. Zhang F. Xue Y. (2025). Mechanisms of anxiety among doctoral students in China. Behav. Sci. 15:105. doi: 10.3390/bs15020105, PMID: 40001736 PMC11851512

[ref10] Bakker A. B. Sanz Vergel A. I. Kuntze J. (2015). Student engagement and performance: a weekly diary study on the role of openness. Motiv. Emot. 39, 49–62. doi: 10.1007/s11031-014-9422-5

[ref11] Bandura A. (1982). Self-efficacy mechanism in human agency. Am. Psychol. 37, 122–147. doi: 10.1037/0003-066X.37.2.122

[ref12] Bandura A. (1986a). Social foundations of thought and action: A social cognitive theory. Englewood Cliffs, NJ: Prentice Hall.

[ref13] Bandura A. (1986b). The explanatory and predictive scope of self-efficacy theory. J. Soc. Clin. Psychol. 4, 359–373. doi: 10.1521/jscp.1986.4.3.359

[ref14] Bandura A. (1997). Self-efficacy: The exercise of control. New York: W.H.Freeman and Company.

[ref15] Bishop R. M. Bieschke K. J. (1998). Applying social cognitive theory to interest in research among counseling psychology doctoral students: a path analysis. J. Couns. Psychol. 45, 182–188. doi: 10.1037/0022-0167.45.2.182

[ref16] Bourner T. Bowden R. Laing S. (2001). Professional doctorates in England. Stud. High. Educ. 26, 65–83. doi: 10.1080/03075070124819

[ref17] Bresó E. Schaufeli W. B. Salanova M. (2011). Can a self-efficacy-based intervention decrease burnout, increase engagement, and enhance performance? A quasi-experimental study. High. Educ. 61, 339–355. doi: 10.1007/s10734-010-9334-6

[ref18] Cai F. Cao Y. Gu Y. Xie X. (2020). A qualitative study on the influencing factors of delayed graduation among Ed.D students. Acad. Degrees Graduate Educ., 46–52. doi: 10.16750/j.adge.2020.03.008

[ref19] Cents-Boonstra M. Lichtwarck-Aschoff A. Denessen E. Aelterman N. Haerens L. (2021). Fostering student engagement with motivating teaching: an observation study of teacher and student behaviours. Res. Pap. Educ. 36, 754–779. doi: 10.1080/02671522.2020.1767184

[ref20] China Education Professional Degree Graduate Education Network. (2024). Overview of education professional degree education. Available online at: https://edm.eduwest.com/viewnews.jsp?id=41

[ref22] Du K. Wang Y. Ma X. Luo Z. Wang L. Shi B. (2020). Achievement goals and creativity: the mediating role of creative self-efficacy. Educ. Psychol. 40, 1249–1269. doi: 10.1080/01443410.2020.1806210

[ref23] Elballah K. A. Gaber S. A. Shahat H. A. Ibrahim A. H. Al Hasan S. A. (2024). Future problem-solving skills and its relationship with research self-efficacy in post-graduate students. Int. J. Learn. Teach. Educ. Res. 23, 180–197. doi: 10.26803/ijlter.23.5.10

[ref24] Erdoğdu M. Y. (2019). The mediating role of school engagement in the relationship between attitude toward learning and academic achievement. Int. J. Educ. Liter. Stud. 7, 75–81. doi: 10.7575/aiac.ijels.v.7n.2p.75

[ref25] Fan W. Williams C. M. (2010). The effects of parental involvement on students’ academic self-efficacy, engagement and intrinsic motivation. Educ. Psychol. 30, 53–74. doi: 10.1080/01443410903353302

[ref26] Feldman D. B. Kubota M. (2015). Hope, self-efficacy, optimism, and academic achievement: distinguishing constructs and levels of specificity in predicting college grade-point average. Learn. Individ. Differ. 37, 210–216. doi: 10.1016/j.lindif.2014.11.022

[ref27] Feng Y. (2015). SPSS 22.0 statistical analysis application textbook: Tsinghua University Press.

[ref28] Fokkens-Bruinsma M. Vermue C. Deinum J. F. Van Rooij E. (2021). First-year academic achievement: the role of academic self-efficacy, self-regulated learning and beyond classroom engagement. Assess. Eval. High. Educ. 46, 1115–1126. doi: 10.1080/02602938.2020.1845606

[ref29] Fong C. J. Patall E. A. Snyder K. E. Hoff M. A. Jones S. J. Zuniga-Ortega R. E. (2023). Academic underachievement and its motivational and self-regulated learning correlates: a meta-analytic review of 80 years of research. Educ. Res. Rev. 41:100566. doi: 10.1016/j.edurev.2023.100566

[ref30] Forester M. Kahn J. H. Hesson-McInnis M. S. (2004). Factor structures of three measures of research self-efficacy. J. Career Assess. 12, 3–16. doi: 10.1177/1069072703257719

[ref31] Fredricks J. A. Blumenfeld P. C. Paris A. H. (2004). School engagement: potential of the concept, state of the evidence. Rev. Educ. Res. 74, 59–109. doi: 10.3102/00346543074001059

[ref32] Fredricks J. A. Filsecker M. Lawson M. A. (2016). Student engagement, context, and adjustment: addressing definitional, measurement, and methodological issues. Learn. Instr. 43, 1–4. doi: 10.1016/j.learninstruc.2016.02.002

[ref33] Froiland J. M. Worrell F. C. (2016). Intrinsic motivation, learning goals, engagement, and achievement in a diverse high school. Psychol. Sch. 53, 321–336. doi: 10.1002/pits.21901

[ref34] Gan Y. Zhang J. Wu X. Gao J. (2024). Chain mediating effects of student engagement and academic achievement on university identification. SAGE Open 14:21582440241226903. doi: 10.1177/21582440241226903

[ref35] Gao Y. Chen H. Wang D. (2020). How high is the delayed graduation rate of doctoral students: an empirical study based on the data of the national school leaving survey in 2017. Graduate Educ. Res. 1, 42–51.

[ref36] Geesa R. L. Brown R. D. McConnell K. R. (2020). Mentoring pathways program for first-year education doctor of education students: perspectives of a program redesigned for sustainability. Mentor. Tutor. 28, 156–175. doi: 10.1080/13611267.2020.1749346

[ref37] Guo C. Fang C. Wang L.. (2020). Analysis of influential factors on deferred study completion of doctoral students in education. J. Graduate Educ. 4, 53–59. doi: 10.19834/j.cnki.yjsjy2011.2020.04.09

[ref38] Han Y. (2024). Structural model of intelligence beliefs, motivational beliefs, academic self-handicapping and academic adjustment in Chinese undergraduate students. BMC Psychol. 12:602. doi: 10.1186/s40359-024-01999-w, PMID: 39473005 PMC11523899

[ref39] Han X. Xu Q. Xiao J. Liu Z. (2024). Academic atmosphere and graduate students’ innovation ability: the role of scientific research self-efficacy and scientific engagement. Eur. J. Psychol. Educ. 39, 1027–1044. doi: 10.1007/s10212-023-00737-x

[ref40] Hodgkin K. Davis S. McInch A. Littlewood J. (2024). Exploring the ‘learner journey’ of students undertaking a professional doctorate in Wales. Res. Post-Compuls. Educ. 29, 408–427. doi: 10.1080/13596748.2024.2371647

[ref41] Honicke T. Broadbent J. Fuller-Tyszkiewicz M. (2020). Learner self-efficacy, goal orientation, and academic achievement: exploring mediating and moderating relationships. Higher Educ. Res. Develop. 39, 689–703. doi: 10.1080/07294360.2019.1685941

[ref42] Honicke T. Broadbent J. Fuller-Tyszkiewicz M. (2023). The self-efficacy and academic performance reciprocal relationship: the influence of task difficulty and baseline achievement on learner trajectory. Higher Educ. Res. Develop. 42, 1936–1953. doi: 10.1080/07294360.2023.2197194

[ref43] Hu Y. Zhou W. (2023). Who gets admitted to Ed.D. Programs? An empirical analysis based on admission data from 31 institutions in China in 2022. Academic Degrees Graduate Educ., 61–69. doi: 10.16750/j.adge.2023.08.009

[ref44] Huang L. Wang D. (2023). Teacher support, academic self-efficacy, student engagement, and academic achievement in emergency online learning. Behav. Sci. 13:704. doi: 10.3390/bs13090704, PMID: 37753982 PMC10525361

[ref45] Jian Z. (2022). Sustainable engagement and academic achievement under impact of academic self-efficacy through mediation of learning agility — evidence from music education students. Front. Psychol. 13:899706. doi: 10.3389/fpsyg.2022.899706, PMID: 35774941 PMC9237460

[ref46] Jonathans P. M. Cahyono B. Y. Widiati U. Kweldju S. (2024). Enhancing factors for doctoral students’ writing self-efficacy: a narrative approach. Indonesian J. Appl. Linguist. 14, 143–156. doi: 10.17509/ijal.v14i1.70397

[ref47] Jones M. (2018). Contemporary trends in professional doctorates. Stud. High. Educ. 43, 814–825. doi: 10.1080/03075079.2018.1438095

[ref48] Jones E. A. Piontek J. Walden L. C. Harrell-Williams L. M. (2024). Development and validation of the sources of research self-efficacy scale. J. Psychoeduc. Assess. 42, 29–45. doi: 10.1177/07342829231204507

[ref49] Kerrigan M. R. Hayes K. W. (2016). Ed.D students’ self-efficacy and interest in conducting research. Int. J. Dr. Stud. 11, 147–162. doi: 10.28945/3413

[ref50] Khuram W. Wang Y. Ali M. Khalid A. Han H. (2023). Impact of supportive supervisor on doctoral students’ research productivity: the mediating roles of academic engagement and academic psychological capital. SAGE Open 13:21582440231185554. doi: 10.1177/21582440231185554

[ref51] Klapp T. Klapp A. Gustafsson J. E. (2024). Relations between students’ well-being and academic achievement: evidence from Swedish compulsory school. Eur. J. Psychol. Educ. 39, 275–296. doi: 10.1007/s10212-023-00690-9

[ref52] Kot F. C. Hendel D. D. (2012). Emergence and growth of professional doctorates in the United States, United Kingdom, Canada and Australia: a comparative analysis. Stud. High. Educ. 37, 345–364. doi: 10.1080/03075079.2010.516356

[ref53] Kuh G. D. (1991). Involving colleges: Successful approaches to fostering student learning and development outside the classroom. San Francisco: Jossey-Bass.

[ref54] Li S. Huang J. Hussain S. Dong Y. (2025). How does supervisor support impact Chinese graduate students’ research creativity through research self-efficacy and intrinsic motivation?–a multi-group analysis. Think. Skills Creat. 55:101700. doi: 10.1016/j.tsc.2024.101700

[ref55] Litson K. Blaney J. M. Feldon D. F. (2021). Understanding the transient nature of stem doctoral students’ research self-efficacy across time: considering the role of gender, race, and first-generation college status. Front. Psychol. 12:617060. doi: 10.3389/fpsyg.2021.617060, PMID: 33574789 PMC7870493

[ref56] Liu H. Li D. (2020). Challenges and adjustment: a study on the status quo of the living conditions of full-time doctoral students of education. J. Graduate Educ. 2, 14–20. doi: 10.19834/j.cnki.yjsjy2011.2020.02.03

[ref57] Livinƫi R. Gunnesch-Luca G. Iliescu D. (2021). Research self-efficacy:a meta-analysis. Educ. Psychol. 56, 215–242. doi: 10.1080/00461520.2021.1886103

[ref58] Luo Q. Chen L. Yu D. Zhang K. (2023). The mediating role of learning engagement between self-efficacy and academic achievement among Chinese college students. Psychol. Res. Behav. Manag. 16, 1533–1543. doi: 10.2147/PRBM.S401145, PMID: 37143904 PMC10153452

[ref59] Mackie S. A. Bates G. W. (2019). Contribution of the doctoral education environment to PhD candidates’ mental health problems: a scoping review. Higher Educ. Res. Develop. 38, 565–578. doi: 10.1080/07294360.2018.1556620

[ref60] Martínez-López Z. Moran V. E. Mayo M. E. Villar E. Tinajero C. (2024). Perceived social support and its relationship with self-regulated learning, goal orientation self-management, and academic achievement. Eur. J. Psychol. Educ. 39, 813–835. doi: 10.1007/s10212-023-00752-y

[ref61] Natriello G. (1984). Problems in the evaluation of students and student disengagement from secondary schools. J. Res. Dev. Educ. 17, 14–24.

[ref62] Northey G. Govind R. Bucic T. Chylinski M. Dolan R. van Esch P. (2018). The effect of “here and now” learning on student engagement and academic achievement. Br. J. Educ. Technol. 49, 321–333. doi: 10.1111/bjet.12589

[ref63] Nyunt G. Brown D. Jensen A. Schaefer C. (2023). Motivations to pursue an Ed. d.in higher education: a qualitative case study. J. Stud. Aff. Res. Pract. 60, 688–701. doi: 10.1080/19496591.2022.2111521

[ref64] Olivier E. Archambault I. De Clercq M. Galand B. (2019). Student self-efficacy, classroom engagement, and academic achievement: comparing three theoretical frameworks. J. Youth Adolesc. 48, 326–340. doi: 10.1007/s10964-018-0952-0 30421327

[ref65] Overall N. C. Deane K. L. Peterson E. R. (2011). Promoting doctoral students’ research self-efficacy: combining academic guidance with autonomy support. Higher Educ. Res. Develop. 30, 791–805. doi: 10.1080/07294360.2010.535508

[ref66] Owusu-Agyeman Y. Mugume T. (2023). Academic adjustment of first year students and their transition experiences: the moderating effect of social adjustment. Tert. Educ. Manag. 29, 189–209. doi: 10.1007/s11233-023-09120-3

[ref67] Pajares F. Schunk D. (2001). The development of academic self-efficacy. Development of Achievement Motivation: Academic Press.

[ref68] Qin H. Li J. Zhang B.. (2025). Academic or utilitarian: how educational doctoral students’ learning motivations influence academic achievement. China Higher Educ. Res. 2, 68–74. doi: 10.16298/j.cnki.1004-3667.2025.02.10

[ref69] Qin C. Song H. (2021). A systematic review of the research on domestic Ed.D education (2000-2020). J. Graduate Educ., 1–9. doi: 10.19834/j.cnki.yjsjy2011.2021.06.01

[ref70] Qin H. Zhang B. Zhou J. (2022). Why do Ed.D. Students generally postpone graduation — a survey based on 27 cultivation units in China. J. High. Educ. 43, 57–69.

[ref71] Robbins S. B. Lauver K. Le H. Davis D. Langley R. Carlstrom A. (2004). Do psychosocial and study skill factors predict college outcomes? A meta-analysis. Psychol. Bull. 130, 261–288. doi: 10.1037/0033-2909.130.2.261, PMID: 14979772

[ref72] San C. K. Guo H. (2023). Institutional support, social support, and academic performance: mediating role of academic adaptation. Eur. J. Psychol. Educ. 38, 1659–1675. doi: 10.1007/s10212-022-00657-2

[ref73] Sanchez-Cardona I. Rodriguez-Montalbán R. Acevedo-Soto E. Lugo K. N. Torres-Oquendo F. Toro-Alfonso J. (2012). Self-efficacy and openness to experience as antecedent of study engagement: an exploratory analysis. Procedia Soc. Behav. Sci. 46, 2163–2167. doi: 10.1016/j.sbspro.2012.05.446

[ref74] Sarstedt M. Ringle C. M. Hair J. F. (2021). “Partial least squares structural equation modeling” in Handbook of market research (Springer), 587–632.

[ref9001] Schaufeli W. B. Salanova M. González-Romá V. Bakker A. B. (2002). The measurement of engagement and burnout: A two sample confirmatory factor analytic approach. J. Happiness Stud. 3, 71–92. doi: 10.1023/A:1015630930326

[ref9003] Schneider M. Preckel F. (2017). Variables associated with achievement in higher education: A systematic review of meta-analyses. Psychol. Bull. 143, 565–600. doi: 10.1037/bul0000098 28333495

[ref76] Schunk D. H. (1991). Self-efficacy and academic motivation. Educ. Psychol. 26, 207–231. doi: 10.1080/00461520.1991.9653133

[ref77] Schunk D. H. (1995). Self-efficacy, motivation, and performance. J. Appl. Sport Psychol. 7, 112–137. doi: 10.1080/10413209508406961

[ref9002] Schunk D. H. Pajares F. (2009). Self-efficacy theory. In Handbook of Motivation at School. (eds.) Wentzel K. R. Wigfield A., New York: Routledge, pp. 35–53.

[ref79] Shao Y. Kang S. (2022). The association between peer relationship and learning engagement among adolescents: the chain mediating roles of self-efficacy and academic resilience. Front. Psychol. 13:938756. doi: 10.3389/fpsyg.2022.938756, PMID: 35992466 PMC9384863

[ref80] Skinner E. A. Kindermann T. A. Furrer C. J. (2009). A motivational perspective on engagement and disaffection: conceptualization and assessment of children’s behavioral and emotional participation in academic activities in the classroom. Educ. Psychol. Meas. 69, 493–525. doi: 10.1177/0013164408323233

[ref81] Sökmen Y. (2021). The role of self-efficacy in the relationship between the learning environment and student engagement. Educ. Stud. 47, 19–37. doi: 10.1080/03055698.2019.1665986

[ref83] Stadtlander L. M. Sickel A. Salter D. (2020). Online doctoral student research and writing self-efficacy in a publishing internship. Higher Learn. Res. Commun. 10, 78–89. doi: 10.18870/hlrc.v10i1.1170

[ref84] Steinmayr R. Meißner A. Weidinger A. F. Wirthwein L. (2014). Academic achievement. Oxford bibliographies in psychology. UK: Oxford University Press.

[ref85] Tao Y. Meng Y. Gao Z. Yang X. (2022). Perceived teacher support, student engagement, and academic achievement: a meta-analysis. Educ. Psychol. 42, 401–420. doi: 10.1080/01443410.2022.2033168

[ref86] Täschner J. Dicke T. Reinhold S. Holzberger D. (2025). “Yes, I can!” a systematic review and meta-analysis of intervention studies promoting teacher self-efficacy. Rev. Educ. Res. 95, 3–52. doi: 10.3102/00346543231221499

[ref87] Terry T. Ghosh R. (2015). Mentoring from different social spheres: how can multiple mentors help in doctoral student success in Ed.D programs? Mentor. Tutor. 23, 187–212. doi: 10.1080/13611267.2015.1072396

[ref88] The Carnegie Project on the Education Doctorate. (2024). The CPED framework. Available at: https://cped.memberclicks.net/the-framework (Accessed January 2, 2025).

[ref89] Van Doorsselaere J. (2024). Factors that support teachers as educational professionals doing doctoral studies: an integrative literature review. Front. Educ. 9, 9:1466631. doi: 10.3389/feduc.2024.1466631

[ref90] Van Rooij E. C. Jansen E. P. van de Grift W. J. (2018). First-year university students’ academic success: the importance of academic adjustment. Eur. J. Psychol. Educ. 33, 749–767. doi: 10.1007/s10212-017-0347-8

[ref91] Vincent C. Tremblay-Wragg É. Déri C. Plante I. Mathieu Chartier S. (2021). How writing retreats represent an ideal opportunity to enhance PhD candidates’ writing self-efficacy and self-regulation. Teach. High. Educ. 28, 1600–1619. doi: 10.1080/13562517.2021.1918661

[ref92] Vizoso C. Arias-Gundín O. Rodríguez C. (2019). Exploring coping and optimism as predictors of academic burnout and performance among university students. Educ. Psychol. 39, 768–783. doi: 10.1080/01443410.2018.1545996

[ref93] Walker D. W. Haley-Mize S. (2012). Content analysis of PhD and Ed.D dissertations in special education. Teach. Educ. Spec. Educ. 35, 202–211. doi: 10.1177/0888406411431168

[ref94] Wang X. Yan L. (2019). Relationship between middle school students’ conscience and academic achievement: the mediating effect of learning engagement. Psychol Explorat 39, 78–83.

[ref95] Willess B. (2023). A mixed methods design exploring factors and experiences of Ed.D. Graduates and time to degree. Doctoral dissertation, California State University, San Bernardino. CSUSB scholar works.

[ref96] Wollast R. Aelenei C. Chevalère J. Van der Linden N. Galand B. Azzi A.. (2023). Facing the dropout crisis among PhD candidates: the role of supervisor support in emotional well-being and intended doctoral persistence among men and women. Stud. High. Educ. 48, 813–828. doi: 10.1080/03075079.2023.2172151

[ref97] Wong Z. Y. Liem G. A. D. (2022). Student engagement: current state of the construct, conceptual refinement, and future research directions. Educ. Psychol. Rev. 34, 107–138. doi: 10.1007/s10648-021-09628-3

[ref98] Wu M. L. (2010). Structural equation model: Operation and application of AMOS. Chongqing: Chongqing University Press.

[ref99] Wu M. (2018). Structural equation modeling: Advanced practice with Amos. Chongqing: Chongqing University Press.

[ref100] Wu Z. Rao C. Lü L.. (2022). A theoretical and practical study on the reform of the UGS collaborative training model for Ed.D. Students. Acad. Degrees Graduate Educ., 33–39. doi: 10.16750/j.adge.2022.03.005

[ref101] Yang Y. Gao J. Ren Z. (2024). Research on quality assurance system of doctor of education. Educ. Sci. 40, 55–62.

[ref102] Zambo R. Zambo D. Buss R. R. Perry J. A. Williams T. R. (2014). Seven years after the call: students’ and graduates’ perceptions of the re-envisioned Ed.D. Innov. High. Educ. 39, 123–137. doi: 10.1007/s10755-013-9262-3

[ref103] Zeng Y. Zhang W. Wei J. Zhang W. (2023). The association between online class-related enjoyment and academic achievement of college students: a multi-chain mediating model. BMC Psychol. 11:349. doi: 10.1186/s40359-023-01390-1, PMID: 37865775 PMC10589956

[ref104] Zhang B. Yin X. Ren Z. (2024). Can perceived social support influence academic achievement of master’s students?— evidence from a university in China. Educ. Inf. Technol. 29, 21449–21475. doi: 10.1007/s10639-024-12693-0, PMID: 40381027

[ref105] Zhao J. Sheng Z. (2022). Empowerment: the new path to improve Ed.D education quality: a case study based on H university. J. Graduate Educ., 68–74. doi: 10.19834/j.cnki.yjsjy2011.2022.03.11

[ref106] Zhou J. Chen F. Qin H. (2023). Study on early warning model of postgraduates’ postponement of Ed.D in China: an empirical analysis based on logistic-fisher analyse. China Higher Educ. Res. 3, 27–33. doi: 10.16298/j.cnki.1004-3667.2023.03.05

[ref107] Zimmerman B. J. (1990). Self-regulated learning and academic achievement: an overview. Educ. Psychol. 25, 3–17. doi: 10.1207/s15326985ep2501_2

[ref108] Zimmerman B. J. (2000). Self-efficacy: an essential motive to learn. Contemp. Educ. Psychol. 25, 82–91. doi: 10.1006/ceps.1999.1016 10620383

[ref109] Zysberg L. Schwabsky N. (2021). School climate, academic self-efficacy and student achievement. Educ. Psychol. 41, 467–482. doi: 10.1080/01443410.2020.1813690

