# Supplementary material for: The influence of research self-efficacy and learning engagement on Ed.D students’ academic achievement
Source: Front Psychol. 2025 Jun 2;16:1562354. doi: 10.3389/fpsyg.2025.1562354 (PMC12168161; doi:10.3389/fpsyg.2025.1562354)
Supplement: Supplementary file 1 [file Supplementary_file_1.pdf]

Appendix

Survey on the Relationship Between Ed.D. Students' Research Self-Efficacy, Learning Engagement, and Academic Achievement

Dear Participant,

I am conducting a research study to explore the relationship between Ed.D. students' research self-efficacy, learning engagement, and academic achievement. The aim of this study is to provide valuable insights and recommendations to enhance academic success. Your input is crucial to the success of this research. Your responses will remain strictly confidential. Please answer the questions honestly based on your personal experiences. This questionnaire is completely anonymous, and all data collected will be used exclusively for academic research purposes. Your privacy will be safeguarded, and no personal information will be disclosed. Participation in this study is entirely voluntary, and you may withdraw at any time without any negative consequences. Thank you very much for your cooperation and support!

Part one: Basic Information

Below is your basic information. Please answer the questions.

1. Your gender:

A. Male B. Female

2. Your current grade:

A. First year B. Second year C. Third year D. Fourth year E.Others

3. Your age:

A. Under 30 B. 31 – 35 C. 36 – 40 D. Over 40

4. Your university:

A. Beijing Normal University B. Shaanxi Normal University

5. Your major:

A. School Curriculum and Teaching

B. Educational Leadership and Management

C. Student Development and Education

D. Other majors

6. The total number of papers you have published so far:

A. 0 B. 1 C. 2 D. 3 E. 4 or more

Part two: The research self-efficacy scale

This section aims to understand your research self-efficacy. Please choose according to your actual situation.

1. I can excellently complete the course tasks assigned by my subject teachers.

A. Completely disagree B. Mostly disagree C. Unsure D. Mostly agree E. Completely agree

2. I am confident that I can achieve good grades in my subject courses.

A. Completely disagree B. Mostly disagree C. Unsure D. Mostly agree E. Completely agree

3. In subject course learning, I have strong self-learning ability.

A. Completely disagree B. Mostly disagree C. Unsure D. Mostly agree E. Completely agree

4. I am capable of thoroughly understanding classic works in my field.

A. Completely disagree B. Mostly disagree C. Unsure D. Mostly agree E. Completely agree

5. In my professional studies, I am able to put forward some valuable ideas.

A. Completely disagree B. Mostly disagree C. Unsure D. Mostly agree E. Completely agree

6. My research ability is relatively strong.

A. Completely disagree B. Mostly disagree C. Unsure D. Mostly agree E. Completely agree

7. I am capable of publishing papers with certain academic value.

A. Completely disagree B. Mostly disagree C. Unsure D. Mostly agree E. Completely agree

8. I am capable of excellently completing the research tasks assigned by my supervisor.

A. Completely disagree B. Mostly disagree C. Unsure D. Mostly agree E. Completely agree

9. I actively apply for research projects and am confident in achieving success.

A. Completely disagree B. Mostly disagree C. Unsure D. Mostly agree E. Completely agree

10. I am able to effectively apply scientific research methods in my studies.

A. Completely disagree B. Mostly disagree C. Unsure D. Mostly agree E. Completely agree

11. I actively seek out research questions and fields of interest and conduct research in those areas.

A. Completely disagree B. Mostly disagree C. Unsure D. Mostly agree E. Completely agree

12. When facing challenges in research activities, I still believe in my research ability.

A. Completely disagree B. Mostly disagree C. Unsure D. Mostly agree E. Completely agree

13. I can keenly identify hot issues in my field, think deeply about them, and express my own views.

A. Completely disagree B. Mostly disagree C. Unsure D. Mostly agree E. Completely agree

14. I have the confidence and ability to publish high-quality journal papers.

A. Completely disagree B. Mostly disagree C. Unsure D. Mostly agree E. Completely agree

15. The theoretical knowledge I have learned is very helpful for my teaching practice.

A. Completely disagree B. Mostly disagree C. Unsure D. Mostly agree E. Completely agree

16. I can relatively effectively apply educational theories in my teaching work.

A. Completely disagree B. Mostly disagree C. Unsure D. Mostly agree E. Completely agree

17. I am confident in managing my work and study time effectively.

A. Completely disagree B. Mostly disagree C. Unsure D. Mostly agree E. Completely agree

18. I believe that pursuing a Ph.D. will better improve my educational and teaching abilities.

A. Completely disagree B. Mostly disagree C. Unsure D. Mostly agree E. Completely agree

Part three: The learning engagement scale

This section mainly aims to understand your level of learning engagement. Please choose according to your actual situation.

1. I often spend a lot of time studying in places like the library, study room, or laboratory.

A. Completely disagree B. Mostly disagree C. Unsure D. Mostly agree E. Completely agree

2. I often read literature in my field and related areas.

A. Completely disagree B. Mostly disagree C. Unsure D. Mostly agree E. Completely agree

3. I frequently actively participate in various academic activities related to my field (such as lectures, conferences, academic salons, research projects, etc.).

A. Completely disagree B. Mostly disagree C. Unsure D. Mostly agree E. Completely agree

4. I am able to balance teaching duties and learning tasks well.

A. Completely disagree B. Mostly disagree C. Unsure D. Mostly agree E. Completely agree

5. I often take courses related to academic research.

A. Completely disagree B. Mostly disagree C. Unsure D. Mostly agree E. Completely agree

6. I often discuss academic issues with classmates or teachers.

A. Completely disagree B. Mostly disagree C. Unsure D. Mostly agree E. Completely agree

7. I have long-term plans and short-term goals for my professional studies.

A. Completely disagree B. Mostly disagree C. Unsure D. Mostly agree E. Completely agree

8. I make daily study plans and arrange study tasks reasonably.

A. Completely disagree B. Mostly disagree C. Unsure D. Mostly agree E. Completely agree

9. I often think about and reflect on my academic research.

A. Completely disagree B. Mostly disagree C. Unsure D. Mostly agree E. Completely agree

10. I follow academic WeChat public accounts and keep up with research topics.

A. Completely disagree B. Mostly disagree C. Unsure D. Mostly agree E. Completely agree

11. I can focus intently during my studies and am rarely influenced by the surrounding environment.

A. Completely disagree B. Mostly disagree C. Unsure D. Mostly agree E. Completely agree

12. I really enjoy the major I am currently studying.

A. Completely disagree B. Mostly disagree C. Unsure D. Mostly agree E. Completely agree

13. I believe that the process of research and learning is fulfilling and enjoyable.

A. Completely disagree B. Mostly disagree C. Unsure D. Mostly agree E. Completely agree

14. I greatly cherish the opportunity to continue learning after starting my career.

A. Completely disagree B. Mostly disagree C. Unsure D. Mostly agree E. Completely agree

15. I can actively cope with difficulties in my academic studies.

A. Completely disagree B. Mostly disagree C. Unsure D. Mostly agree E. Completely agree

16. I rarely feel anxious about not completing my study or research tasks.

A. Completely disagree B. Mostly disagree C. Unsure D. Mostly agree E. Completely agree

17. I hope to improve my teaching methods through my Ed.D. studies.

A. Completely disagree B. Mostly disagree C. Unsure D. Mostly agree E. Completely agree

Part four: The academic achievement scale

This section mainly aims to understand your academic achievement. Please choose according to your actual situation.

1. Through my studies, I am now able to apply the research methods I have learned to analyze data.

A. Completely disagree B. Mostly disagree C. Unsure D. Mostly agree E. Completely agree

2. Through my studies, I am now able to make innovations based on existing research findings.

A. Completely disagree B. Mostly disagree C. Unsure D. Mostly agree E. Completely agree

3. Through my studies, I am now able to apply the knowledge I have learned to analyze problems.

A. Completely disagree B. Mostly disagree C. Unsure D. Mostly agree E. Completely agree

4. Through my studies, I am now able to apply the professional knowledge I have learned to practice and use it to solve problems in teaching.

A. Completely disagree B. Mostly disagree C. Unsure D. Mostly agree E. Completely agree

5. Through my studies, I am now able to use various methods to collect data or materials for writing papers.

A. Completely disagree B. Mostly disagree C. Unsure D. Mostly agree E. Completely agree

6. Through the training in writing course papers and small essays, I have developed a good ability to write papers.

A. Completely disagree B. Mostly disagree C. Unsure D. Mostly agree E. Completely agree

7. Through my studies, I am better able to conduct research design, and I can keenly identify problems, raise questions, and find solutions.

A. Completely disagree B. Mostly disagree C. Unsure D. Mostly agree E. Completely agree

8. During my Ed.D. studies, I have mastered the cutting-edge knowledge in my field.

A. Completely disagree B. Mostly disagree C. Unsure D. Mostly agree E. Completely agree

9. During my Ed.D. studies, I have mastered the fundamental theoretical knowledge in my field.

A. Completely disagree B. Mostly disagree C. Unsure D. Mostly agree E. Completely agree

10. Through my professional studies, I am familiar with the classic works and their ideas in my field.

A. Completely disagree B. Mostly disagree C. Unsure D. Mostly agree E. Completely agree

11. Through extensive literature reading, I am familiar with the existing research in my field of study.

A. Completely disagree B. Mostly disagree C. Unsure D. Mostly agree E. Completely agree

12. Through my professional studies, my knowledge and perspective have been broadened.

A. Completely disagree B. Mostly disagree C. Unsure D. Mostly agree E. Completely agree

13. I have mastered a systematic and comprehensive theory of education and teaching.

A. Completely disagree B. Mostly disagree C. Unsure D. Mostly agree E. Completely agree

Thank you once again for your patience and support!
